# DEAE-Dextran Enhances the Lentiviral Transduction of Primary Human Mesenchymal Stromal Cells from All Major Tissue Sources Without Affecting Their Proliferation and Phenotype

**DOI:** 10.1007/s12033-022-00549-2

**Published:** 2022-08-23

**Authors:** Francesco Amadeo, Vivien Hanson, Patricia Murray, Arthur Taylor

**Affiliations:** 1 Cellular Therapies Laboratory, NHS Blood and Transplant, Liverpool, UK; 2 Department of Molecular Physiology and Cell Signalling, University of Liverpool, Liverpool, UK

**Keywords:** Mesenchymal stromal cells, Polycations, Lentivirus, Transduction, Reporter genes

## Abstract

Genetic engineering of mesenchymal stromal cells (MSCs) is a tool widely used to explore MSC properties in vitro and in vivo. Lentiviral infection with the use of polycations as an adjuvant is a method that is commonly used to generate stably transduced cells. However, it is known that some polycations can negatively affect primary MSCs and to date, no study has explored the effect of different polycations on the transduction efficiency and properties of all main types of MSCs, namely those derived from umbilical cord, bone marrow and adipose tissue. Here we explore a range of polycations, using transduction protocols with and without spinoculation, to produce stably transduced MSCs from these three tissue sources. We identified that an overnight incubation with diethylaminoethyl-dextran (DEAE-Dextran) is the protocol associated with the best transduction efficiency without compromising the viability of the cells, and which worked consistently with lentiviral particles encoding for different transgenes. Transduced and sorted MSC populations revealed no significant changes in proliferation, morphology and expression of MSC markers compared to naïve MSCs. Following this study, we conclude that DEAE-Dextran is a polycation that can be successfully used to enhance the transduction of MSCs from all major tissue sources.

**Supplementary Information:**

The online version contains supplementary material available at 10.1007/s12033-022-00549-2.

## Introduction

Mesenchymal stromal cells (MSCs) are multipotent cells showing great promise in different pre-clinical studies and currently used in many clinical trials [1, 2]. Several properties and mechanisms of actions of these cells are still not completely understood and genetic engineering is a tool that can be used to gain a better understanding of their properties in vitro [3], help to investigate what happens to them when administered in vivo [4, 5], or even as a strategy to improve their therapeutic efficacy [6].

Lentiviral vectors (LV) are widely used as gene delivery vectors for various research applications because of their ability to infect both dividing and non-dividing cells. The interaction between the lentiviral particle and the cell of interest is receptor independent [7] and can be reduced or prevented by the presence of strong electrostatic repulsion between the negatively charged cell and the envelope of the virus [8, 9].

The addition of polycations, positively charged molecules, can reduce the charge of cell membranes, and increase the chance of an interaction between the lentivirus and the cell surface [9]. Polybrene (Pb) is the most commonly used polycation in lentiviral transduction and is associated with a very high transduction efficiency [10, 11]. Nevertheless, it has also been reported to have negative effects on some types of cell [12–14]. In particular, its use for the transduction of human endometrium-derived mesenchymal stromal cells has been described to negatively affect their proliferation, migration ability and differentiation potential [14]. Other polycations, such as diethylaminoethyl-dextran (DEAE-dextran) and protamine sulphate (Ps) have also been successfully used to transduce different cell lines [9, 15].

Another method reported to increase the binding of retroviruses to the surface of the cells is spinoculation [16–18]. Although various mechanisms have been proposed [16, 17], it is still unclear what process is responsible for this enhancement. During spinoculation, the cells are centrifuged at low speed (below 2000 g) in the presence of the virus. Importantly, it has been reported that spinoculation together with an optimal polycation can synergistically increase transduction efficiency [15].

A transduction method that is universally efficacious across all major types of MSC would facilitate research on their properties and mechanisms of action but to date, no studies have assessed how different protocols affect MSCs from different tissue sources and across different donors. Importantly, any protocol for the genetic modification of MSCs must not alter their properties, unless that is the specific intention such as in a gene knockdown study, as this would compromise the research findings. In this work, we optimise a transduction protocol with the goal of obtaining genetically engineered umbilical cord (UC), bone marrow (BM) and adipose-derived (A) MSCs that share the same properties as the unmodified (naïve) cells. We identify that a protocol based on the use of DEAE-dextran is capable of producing stable transduced mesenchymal stromal cell populations without affecting their properties.

## Materials and Methods

### Cell Culture

Human MSCs from three different tissue sources were used. Umbilical cord (UC) MSCs were obtained from the NHS Blood and Transplant (NHSBT, Liverpool, UK), licenced under the UK’s Human Tissue Act (HTA). The cells were isolated according to NHSBT good manufacturing practice (GMP) procedures: the tissue was halved horizontally, chopped into big pieces and cultured undisturbed for 7 days. The pieces were then removed and the adherent cells were expanded for two passages under standard tissue culture conditions. Bone marrow (BM) MSCs were provided by the University of Galway (Ireland), after being purchased from Lonza (Basel, Switzerland). Adipose derived (A) MSCs were provided by the University of Heidelberg (Germany), after being isolated from lipoaspirates harvested with informed consent. Briefly, the tissue was digested with a solution of NB4 collagenase (Serva/Nordmark) under gentle agitation for 30 min. After a straining step to remove the undigested tissue, the cells were centrifuged, resuspended in complete medium and expanded under standard tissue culture conditions. The Mannheim Ethics Commission II approved the study (vote 2011-215N-MA). Cells from three different donors were obtained for each tissue source, leading to a total of 9 MSC samples. The cells were cryopreserved and shipped to the Department of Molecular Physiology and Cell Signalling of the University of Liverpool, where all the cells were cultured following standard mammalian tissue culture protocols. The cells were grown in MEM-α containing GlutaMAX (Gibco) and supplemented with 10% foetal bovine serum (FBS; Gibco) and kept at 37 °C in a humidified incubator, with 5% CO2.

### Transduction

In the set of experiments aimed to explore different transduction protocols we infected the 3 types of MSCs with pHIV-Luc2-ZsGreen (Supplementary Fig. 1a) and a multiplicity of infection (MOI) of 5. The pHIV-Luc2-ZsGreen vector was a gift from Bryan Welm (Addgene plasmid #39196). We seeded the cells at a density of 5 × 10^3^ cells/cm^2^ and transduced them 4 h later in the presence of three different polycations: polybrene (Pb, 8 μg/mL), protamine sulphate (Ps, 20 μg/mL) or DEAE-dextran (6 μg/mL). MSCs transduced without any adjuvant served as control for the basal transduction efficiency. To evaluate a possible effect of the polycations themselves on the cells, we also incubated the cells with either Pb, Ps or DEAE-dextran alone. Finally, we used MSCs alone (without any polycation or LV) as controls. We transduced the cells under two conditions: (a) overnight incubation at 37 °C (“static” condition), replacing the medium containing the LV/adjuvant after 16 h or (b) centrifugation of the cells for 1 h at 750 g and room temperature, followed by incubation for 1 h at 37 °C, and then replacing the medium containing the LV/adjuvant (“centrifuged” condition). We assessed the transduction efficiency 7–10 days post transduction via flow cytometry using a FACScalibur (BD Biosciences) and used the CellTiter-Glo® Luminescent Cell Viability Assay (#G7571, Promega) to evaluate the effect of the transduction on the viability and proliferation of the cells.

Once we identified the optimal transduction protocol, we applied it for the transduction with 4 other different lentiviral particles. We seeded MSCs at a density of 5 × 10^3^ cells/cm^2^ and transduced them with the following lentiviral particles (Supplementary Fig. 1b): (i) pHIV-eGFP (a gift from Bryan Welm & Zena Werb; Addgene plasmid #21373), (ii) pHIV-dTomato (a gift from Bryan Welm; Addgene plasmid #21374), (iii) pCDH-EF1α-Luc2-P2A-tdTomato (a gift from Kazuhiro Oka; Addgene plasmid #72486), (iv) pLV-mCherry (a gift from Pantelis Tsoulfas, Addgene plasmid #36084) in individual experiments. All transductions were carried out using a MOI of 5 in the presence or absence of DEAE-dextran (6 μg/mL) with the static protocol. We assessed the transduction efficiency via flow cytometry after 7 days of culture.

In the set of experiments aimed to evaluate the effect of the transduction on the proliferation, expression of MSC markers and morphology of the cells, we transduced the MSCs with the pHIV-Luc2-ZsGreen construct (MOI of 5) in the presence of 6 μg/mL of DEAE-dextran applying the static protocol. Following expansion, we sorted the cells based on the ZsGreen expression using a FACSaria II (BD Biosciences). Then, we compared these cells to untransduced controls in terms of proliferation, morphology and expression of MSC markers. Table 1 summarises the passage of each donor sample at each study step.

**Table 1:** UC, BM and A-MSCs samples and the respective passages at which they were transduced, sorted and used for the experiments

Cell type	Donor ID	Transduction passage	Sorting passage	Proliferation analysis from	Characterisation^a^
UC-MSCs	UC-01	P5	P6	P7	P8
	UC-02	P5	P6	P7	P8
	UC-03	P5	P6	P7	P8
BM-MSCs	BM-01	P3	P4	P5	P6
	BM-02	P5	P6	P7	P8
	BM-03	P3	P4	P5	P6
A-MSCs	A-01	P5	P6	P7	P8
	A-02	P5	P6	P7	P8
	A-03	P5	P6	P7	P8

^a^Characterisation includes flow cytometry, morphological and BLI analysis

### Proliferation and Doubling Time

After sorting and expansion for one passage, Luc2-ZsGreen^+^ and naïve MSCs were seeded at their optimal seeding density (3 × 10^2^ cells/cm^2^ for adipose cells and 3 × 10^3^ cells/cm^2^ for umbilical cord and bone marrow cells) and expanded until 60–90% confluence for at least two passages (Table 1) from where we calculated the doubling time using the following equation:

$$Td=\frac{t}{{Log}_{2}(\frac{{N}_{t}}{{N}_{0}})}$$

where *Td* is the doubling time, *N*_*t*_ is the number of cells at time *t* and *N*_0_ is the number of cells seeded, from which the number of doublings was calculated based on the time the cells had been in culture.

### MSC Markers

MSC marker expression was assessed via flow cytometry by staining the cells with anti CD44 (APC, #130-113-893, Miltenyi Biotec), anti CD45 (APC, #130-113-676, Miltenyi Biotec), anti CD73 (APC, #130-097-945, Miltenyi Biotec), anti CD90 (APC, #130-117-534, Miltenyi Biotec), anti CD105 (APC, #130-099-125, Miltenyi Biotec), IgG1 mouse isotype (APC, #130-113-758, Miltenyi Biotec) or IgG2 mouse isotype (APC, #130-113-831, Miltenyi Biotec) according to the manufacturer’s instructions. An extra vial of each cell population was used as unstained control. We acquired the data with a FACScalibur (BD Biosciences) flow cytometer and we analysed a minimum of 10^4^ events for each marker.

### Morphological Analysis of pHIV Luc2 ZsGreen Transduced MSCs

We cultured MSCs with complete medium overnight. After 16 h we fixed the cells with formaldehyde (4% w/v in PBS, pH 7.4) for 20 min at room temperature (RT), then we washed the cells with PBS, permeabilised them with 0.1% (v/v) Triton X-100 in PBS and incubated them with Alexa Fluor 594 Phalloidin (#A12381, ThermoFisher) [165 nM] in PBS with 1% (w/v) bovine serum albumin (BSA) for 1 h at RT. We used 4′,6 diamidino-2-phenylindole (DAPI) [143 nM] as a counter stain for the nuclei. We acquired the fluorescence images with a Leica DM2500 microscope coupled to a DFC350 FX camera. Finally, we used ImageJ to perform the data analysis: the perimeter the cells was delineated manually, based on the phalloidin staining, and the software was used to calculate the cells’ area and circularity.

### Bioluminescence Imaging of pHIV Luc2 ZsGreen Transduced MSCs

We used the IVIS Spectrum system (Perkin Elmer) to characterise the total flux (photons/second) emitted by the Luc2-ZsGreen^+^ MSCs. We plated the cells at increasing densities, from 156 to 2 × 10^4^ cells/well, in optical bottom 96-well plates with black walls (#165,305, ThermoFisher) and a technical triplicate. Untransduced cells were used as negative control. We allowed the cells to adhere for 3 h and then we added D-Luciferin at a final concentration of 5.12 mM in a final volume of 100 µL per well. We collected the data using the IVIS immediately after the addition of the substrate, acquiring the signal generated using an open filter. The acquired signal was always normalised to radiance (photons/second/centimeter^2^/steradian) and analysed using the region of interest (ROI) tool of the IVIS software (Living Image v 4.5.2) to obtain the flux.

### Statistical Analysis

All values in graphs are represented as mean ± standard deviation, unless specified differently in the figure legend. The statistical analysis was performed using the GraphPad Prism software. The type of statistical test and the number of replicates included in the analyses are indicated in the figure legends.

## Results

### Optimisation of a Lentiviral Transduction Protocol

The cells were infected with a lentiviral particle encoding for firefly luciferase (FLuc) and a green fluorescent protein, ZsGreen, whose expression was used to determine the transduction efficiency via flow cytometry. In parallel, an ATP assay was used to investigate the effect of the different protocols on the proliferation of the cells.

**Fig. 1:**
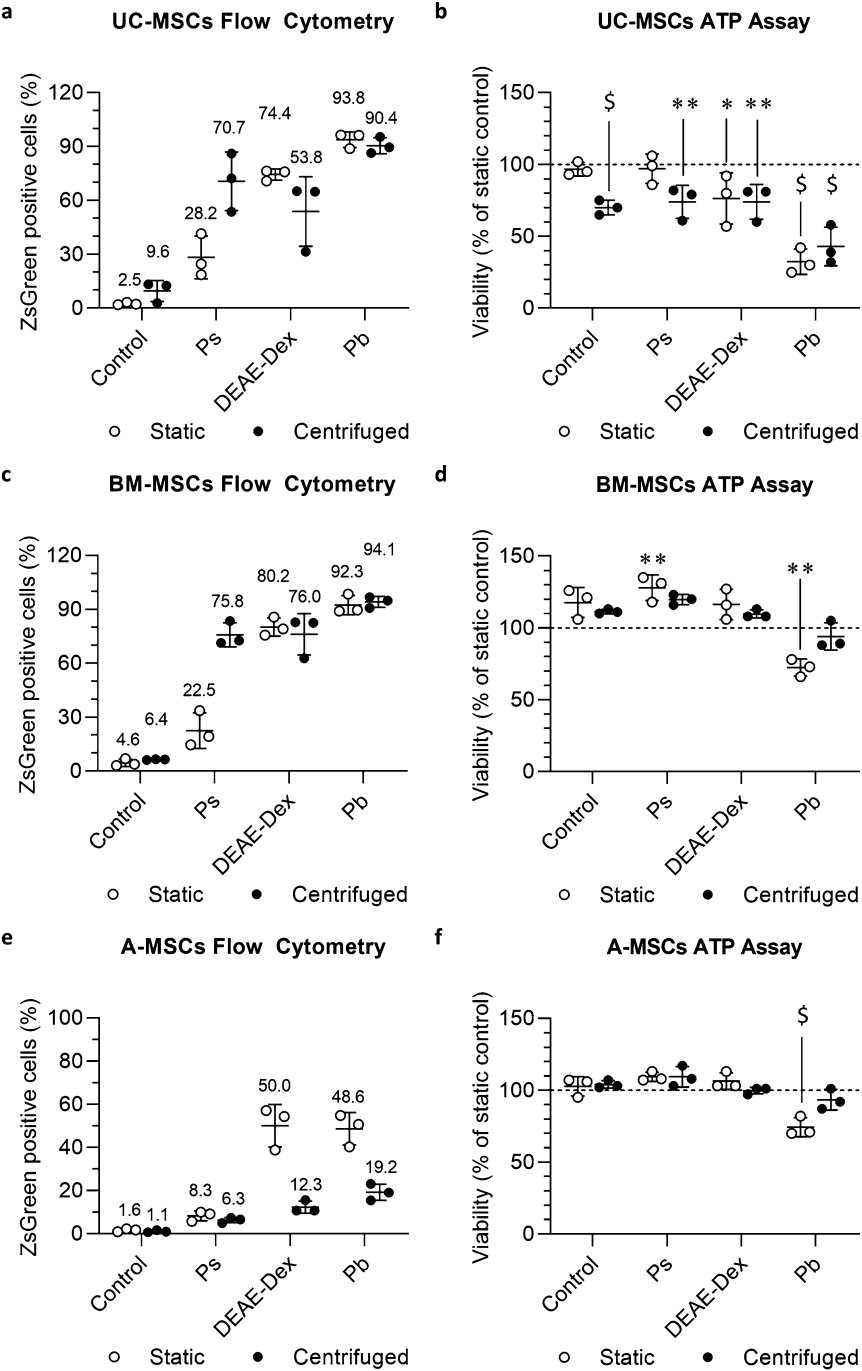
Static DEAE-dextran is a valid alternative to Pb for the transduction of mesenchymal stromal cells. **a** Percentage of transduced UC cells expressing ZsGreen as evaluated via flow cytometry. **b** Levels of ATP produced by transduced UC-MSCs after 7 days of culture following the application of the different protocols. **c** Percentage of transduced BM cells expressing ZsGreen as evaluated via flow cytometry. **d** Levels of ATP produced by transduced BM-MSCs after 10 days of culture following the application of the different protocols. **e** Percentage of transduced A-MSCs expressing ZsGreen as evaluated via flow cytometry. **f** Levels of ATP produced by transduced A-MSCs cells after 7 days of culture following the application of the different protocols. All data are displayed as mean ± SD with *n* = 3 individual donors for each tissue source. For the ATP assay each donor sample was measured in triplicate and averaged. Data are normalised to the untransduced control group in static condition and expressed as percentage. Charts display only the data from transduced cells. ATP assay data from untransduced cells are displayed in Supplementary Fig. 5. Statistical analysis applies only to the ATP assay and was performed on the whole dataset as shown in supplementary Fig. 5a–c: Three-way ANOVA with Dunnett post hoc comparison test against the respective untransduced static control; * *p* < 0.05, ** *p* < 0.01, $ *p* < 0.0001

UC-MSC transduction efficiency in the presence of Pb was high with both the static (93.8 ± 4.4%) and the centrifuged (90.4 ± 4.5%) protocol (Fig. 1a, fluorescence images of the cells are shown in Supplementary Fig. 2). However, the ATP assay of transduced cells in the presence of Pb revealed a strong reduction in the proliferation of the transduced cells, which was only 32.3% (± 8.6%) and 44.1% (± 12.5%) of the control in static and centrifuged conditions, respectively (data of untransduced UC-MSCs are shown in Supplementary Fig. 5a). Cells transduced in the presence of DEAE-dextran resulted in 74.4% (± 3.1%) and 53.8% (± 19.4%) of positive cells (Fig. 1a) in static and centrifuged conditions and were, respectively, associated with a proliferation reduced to 79.3% (± 17.8%) and 74.1% (± 12.2%) (Fig. 1b). Finally, in the presence of Ps (Fig. 1a), 28.2% (± 11.9%) and 70.7% (± 16.3%) of the cells were positive for ZsGreen in static and centrifuged conditions, respectively. These conditions were associated with a reduction in proliferation to 96.9% (± 10.1%) and to 74.0% (± 11.4%) (Fig. 1b).

Similar results were obtained with BM-MSCs, where the transduction in the presence of Pb was associated with 92.3 ± 5.3% and 94.1 ± 3.0% of the cells expressing the ZsGreen protein following the application of the static and the centrifuged protocols, respectively (Fig. 1c, fluorescence images of the cells are shown in Supplementary Fig. 3). Like for UC-MSCs, the presence of Pb resulted in a reduction in the proliferation of the transduced cells to 72.3 ± 6.0% of the control in static condition but did not really affect the growth of the cells when the Pb was combined with a centrifugation step (94.0 ± 9.5%, Fig. 1d). Cells transduced in the presence of DEAE-dextran resulted in 80.2% (± 5.1%) and 76.0% (± 11.5%) of positive cells (Fig. 1c) in static and centrifuged conditions, respectively. Surprisingly, the presence of the DEAE-dextran seems to have increased the proliferation of the transduced cells to 116.3% (± 10.5%) and to 109.7% (± 2.9%), in static and centrifuged conditions, respectively (Fig. 1d). This increase in the proliferation was also observed when the cells were incubated without the lentiviral particles and with the DEAE-dextran only (120.7 ± 10.8% in static and 106.0 ± 3.0% in centrifuged condition, Supplementary Fig. 5b). Finally, in the presence of Ps (Fig. 1c), 22.5% (± 10.0%) and 75.8% (± 6.7%) of the cells were positive for ZsGreen in static and centrifuged conditions, respectively. The presence of the Ps was also associated with an increase in proliferation of transduced cells both in static (128 ± 8.9%) and centrifuged conditions (119.7 ± 3.5%) (Fig. 1d).

For A-MSCs, transduction in the presence of Pb in static and centrifuged conditions was associated with only 48.6 ± 7.5% and 19.2 ± 3.8% of cells effectively transduced, respectively (Fig. 1e, fluorescence images of the cells are shown in Supplementary Fig. 4). Cells transduced in the presence of DEAE-dextran resulted in 50.0% (± 9.9%) and 12.3% (± 2.8%) of positive cells (Fig. 1e) in static and centrifuged conditions, respectively. The transduction with Ps resulted in only 8.3% (± 2.3%) and 6.3% (± 1.3%) of positive cells (Fig. 1e). The ATP assay revealed that the presence of Pb affected the proliferation of the transduced cells following the application of the static protocol (74.4 ± 6.8%), whilst none of the other conditions influenced ATP levels (Fig. 1f, Supplementary Fig. 5c).

A three-way ANOVA analysis performed on the whole dataset of each cell type (Supplementary Fig. 5) revealed that the main impact on the viability for BM and A-MSCs is related to the presence of the polycations (*p* < 0.0001 and *p* = 0.001, respectively). On the other hand, the UC-MSCs were found to be susceptible not only to the presence of polycations (*p* < 0.001), but also to the transduction (*p* < 0.05) and to the centrifugation (*p* < 0.01).

### Validation of the Selected Protocol: DEAE-Dextran in Static Condition

The use of DEAE-dextran with overnight incubation in static conditions was selected as the optimal transduction procedure. This is because it resulted in a good transduction efficiency for all the MSCs tested (74.4% for UC-MSCs, 80.2% for BM-MSCs and 50.0% for A-MSCs) without overtly affecting the proliferation/viability of the cells. This protocol was then applied to transduce the three types of MSCs with different lentiviral particles to assess if the transduction efficiency with this protocol is influenced by the lentiviral construct itself.

Supplementary Fig. 6 displays representative fluorescence images of all three MSCs transduced with the four lentiviral particles in the absence or presence of DEAE-dextran. Regardless of the lentiviral particle, all the cell types display a low transduction efficiency when transduced without polycations. In contrast, for all four lentiviral particles, the presence of DEAE-dextran leads to an increase in the number of transduced cells and in the intensity of signal in all three MSCs (Supplementary Fig. 6a–c). This increase is particularly noticeable for the pLV-mCherry and for the pHIV-dTomato, whilst less evident for the pHIV-eGFP and for the pCDH-EF1α-Luc2-P2A-tdTomato (especially for the BM-MSCs, Supplementary Fig. 6b).

**Fig. 2:**
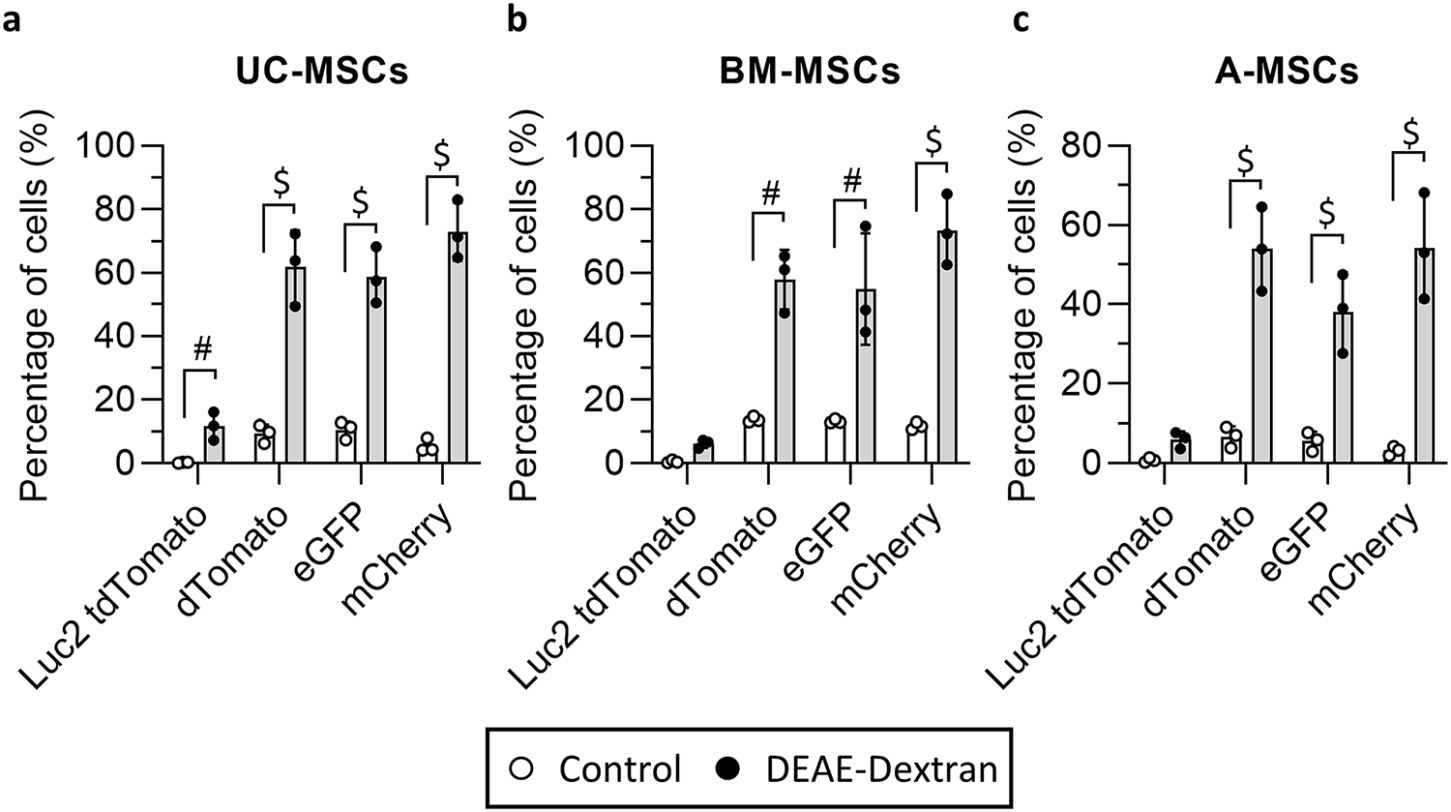
Application of the DEAE-dextran protocol to transduce MSCs with four different lentiviral particles. Percentage of transduced and control UC- (**a**), BM- (**b**) and A-MSCs (**c**) expressing either the tdTomato, the dTomato, the eGFP or the mCherry protein as measured via flow cytometry. Data are displayed as mean ± SD, *n* = 3 individual donors for each tissue source. Two-way ANOVA analysis with Sidak’s multiple comparison post hoc test; $ *p* ≤ 0.0001 and # *p* ≤ 0.0005

The flow cytometry analysis (Fig. 2) of these cells revealed that UC-MSCs displayed a statistically significant increase in the fraction of transduced cells when infected in the presence of the DEAE-dextran for all the lentiviral particles (Fig. 2a). The proportion of cells positive for the transgene increased from 0.3 ± 0.18% to 11.7 ± 4.4% for the pCDH-EF1α-Luc2-P2A-tdTomato (*p* = 0.0005), from 9.4 ± 2.8% to 61.8 ± 11.6% for the pHIV-dTomato (*p* < 0.0001), from 10.5 ± 2.7% to 58.7 ± 8.9% for the pHIV-eGFP (*p* < 0.0001) and from 5.5 ± 2.2% to 72.9 ± 9.2% for the pLV-mCherry (*p* < 0.0001, Fig. 2a). A two-way ANOVA analysis also revealed that both the presence of DEAE-dextran and the specific lentiviral construct used have a statistically significant impact on the transduction efficiency (*p* < 0.01 and *p* < 0.0001, respectively).

Similar results were obtained with the BM-MSCs (Fig. 2b), where the proportion of transduced cells increased from 0.49 ± 0.46% to 6.3 ± 1.3% for the pCDH-EF1α-Luc2-P2A-tdTomato (no statistically significant difference), from 13.7 ± 0.9% to 57.8 ± 9.4% for the pHIV-dTomato (*p* < 0.0005), from 13.2 ± 0.7% to 54.8 ± 17.6% for the pHIV-eGFP (*p* = 0.0005) and from 11.7 ± 1.1% to 73.2 ± 11.2% for the pLV-mCherry (*p* < 0.0001, Fig. 2b). A two-way ANOVA analysis confirmed the statistically significant effect of the DEAE-dextran and of the lentiviral construct on the transduction efficiency (*p* < 0.05 and *p* = 0.0001, respectively).

Lastly, the adipose-derived cells showed a transduction behaviour comparable to the other two MSCs (Fig. 2c), with an increase in the percentage of transduced cells from 0.8 ± 0.54% to 5.9 ± 2.1% for the pCDH-EF1α-Luc2-P2A-tdTomato (no statistically significant difference), from 6.7 ± 2.6% to 54.0 ± 10.6% for the pHIV-dTomato (*p* < 0.0001), from 5.6 ± 2.4% to 38.1 ± 10.0% for the pHIV-eGFP (*p* < 0.0001) and from 3.2 ± 1.2% to 54.2 ± 13.4% for the pLV-mCherry (*p* < 0.0001, Fig. 2c). The two-way ANOVA analysis identified a statistically significant impact of the DEAE-dextran and of the type of lentiviral particle on the transduction efficiency (*p* < 0.05 and *p* < 0.0001, respectively).

### Characterisation of the MSCs Transduced with the DEAE-Dextran Static Protocol

To determine whether the transduction procedure had an impact on the properties of the different MSCs, all cells were transduced with the pHIV-Luc2-ZsGreen construct and sorted to obtain a population approximately 100% positive for the transgenes. Then, transduced and untransduced cells were compared in terms of doubling time, expression level of MSC markers and morphology (area and circularity of the cells).

**Fig. 3:**
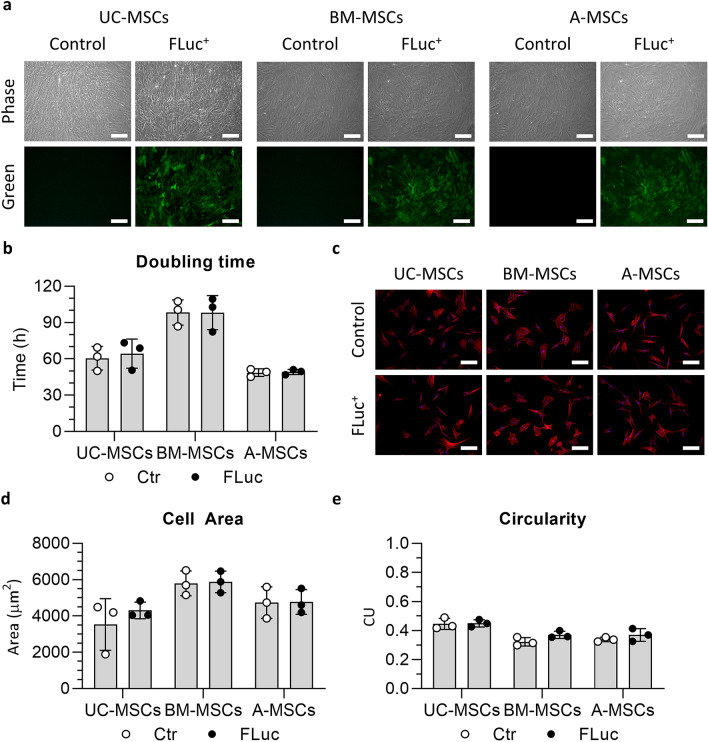
Luc2-ZsGreen^+^ MSCs display similar properties to untransduced cells. **a** Representative phase contrast and green fluorescence images of UC, BM and A-MSCs after sorting compared to their untransduced counterparts. Scale bar = 200 µm. **b** Average doubling times of transduced and untransduced cells. Data are displayed as mean ± SD, n = 3 individual donors. Two-way ANOVA analysis with Sidak’s multiple comparisons post hoc test. **c** Fluorescence images of cells 16 h after seeding stained with phalloidin (f-actin, red) and DAPI (nuclei, blue) acquired at 100x. Scale bar = 200 μm. **d–e** Average cell area (**d**) and circularity (**e**) of transduced and untransduced MSCs. Full dataset shown in Supplementary Fig. 8. FLuc = Luc2-ZsGreen^+^ cells. Data are displayed as mean ± SD, *n* = 3 individual donors. Two-way ANOVA analysis with Sidak’s multiple comparisons post hoc test. Abbreviations: CU = circular unit (1 = perfect circle, 0 = elongated polygon)

Figure 3a shows phase contrast images of control and Luc2-ZsGreen^+^ UC, BM and A-MSCs cells and the respective green fluorescence images (ZsGreen). The morphology of the different transduced cells looks comparable to controls (Fig. 3a). The analysis of the cumulative doublings revealed that the transduced cells behaved similarly to the untransduced ones for at least 2 passages (Supplementary Fig. 7a–c) and no statistically significant difference in the doubling time was observed between transduced and untransduced cells (Fig. 3b and Supplementary Fig. 7d).

The flow cytometry analysis of the expression of ZsGreen was performed two passages after sorting (Table 1) to evaluate any loss of the reporter gene following the expansion. All the transduced populations resulted in a positivity level of over 98.5% (Table 2), underlining the stability of the transduction. Furthermore, the flow cytometry analysis of surface markers revealed a similar level of expression of positive markers (CD44, CD73, CD90 and CD105) and the lack of CD45 negative marker (Table 2) in both transduced and untransduced cells. Only MSCs from one A-MSC donor (A-02) resulted in an increase in the percentage of cells positive for CD45, from 9.28% to 75.52% (Table 2).

**Table 2:** Percentage of Luc2-ZsGreen cells expressing the ZsGreen protein and percentage of untransduced controls and transduced cells positive to CD44, CD45, CD73, CD90 and CD105

Donor ID	Positive cells (ZsGreen)	CD45 (%)	CD44 (%)	CD73 (%)	CD90 (%)	CD105 (%)
UC-01 Ctr	N/A	1.75	99.20	99.88	99.95	99.83
UC-01 FLuc	98.62%	2.68	99.75	99.94	99.95	99.79
UC-02 Ctr	N/A	2.96	99.20	99.77	100.00	99.57
UC-02 FLuc	99.08%	4.59	99.65	99.80	99.95	99.49
UC-03 Ctr	N/A	1.84	97.37	99.80	99.96	99.95
UC-03 FLuc	99.36%	5.01	95.30	99.74	99.93	99.81
BM-01 Ctr	N/A	6.23	99.85	96.84	100.00	98.49
BM-01 FLuc	99.84%	6.96	99.97	99.72	99.99	99.64
BM-02 Ctr	N/A	2.17	99.91	99.74	99.83	97.64
BM-02 FLuc	99.49%	1.98	99.96	98.99	99.86	93.58
BM-03 Ctr	N/A	1.50	99.91	99.90	99.84	99.04
BM-03 FLuc	99.86%	1.34	100.00	99.88	99.70	98.63
A-01 Ctr	N/A	9.11	99.81	99.89	99.98	98.82
A-01 FLuc	99.04%	4.07	100.00	99.50	99.99	93.79
A-02 Ctr	N/A	9.28	99.61	99.53	99.99	98.61
A-02 FLuc	99.45%	75.52	99.98	99.39	99.98	99.87
A-03 Ctr	N/A	12.96	99.94	99.84	100.00	99.96
A-03 FLuc	99.40%	11.20	99.88	99.65	99.97	99.75

*FLuc* Luc2-ZsGreen^+^ cells

Figure 3c shows representative fluorescent images of transduced and untransduced cells stained with phalloidin and DAPI. Morphological analysis of the transduced cells revealed no statistically significant difference in the area (Fig. 3d) or circularity (Fig. 3e) of transduced MSCs compared to untransduced cells. MSCs from one UC-MSC donor (UC-02) displayed an increase in the area (Supplementary Fig. 8a), with no impact on the circularity (Supplementary Fig. 8b) and MSCs from one A-MSC donor (A-03) showed a slight increase in the circularity (Supplementary Fig. 8f), with no difference in the area (Supplementary Fig. 8e).

**Fig. 4:**
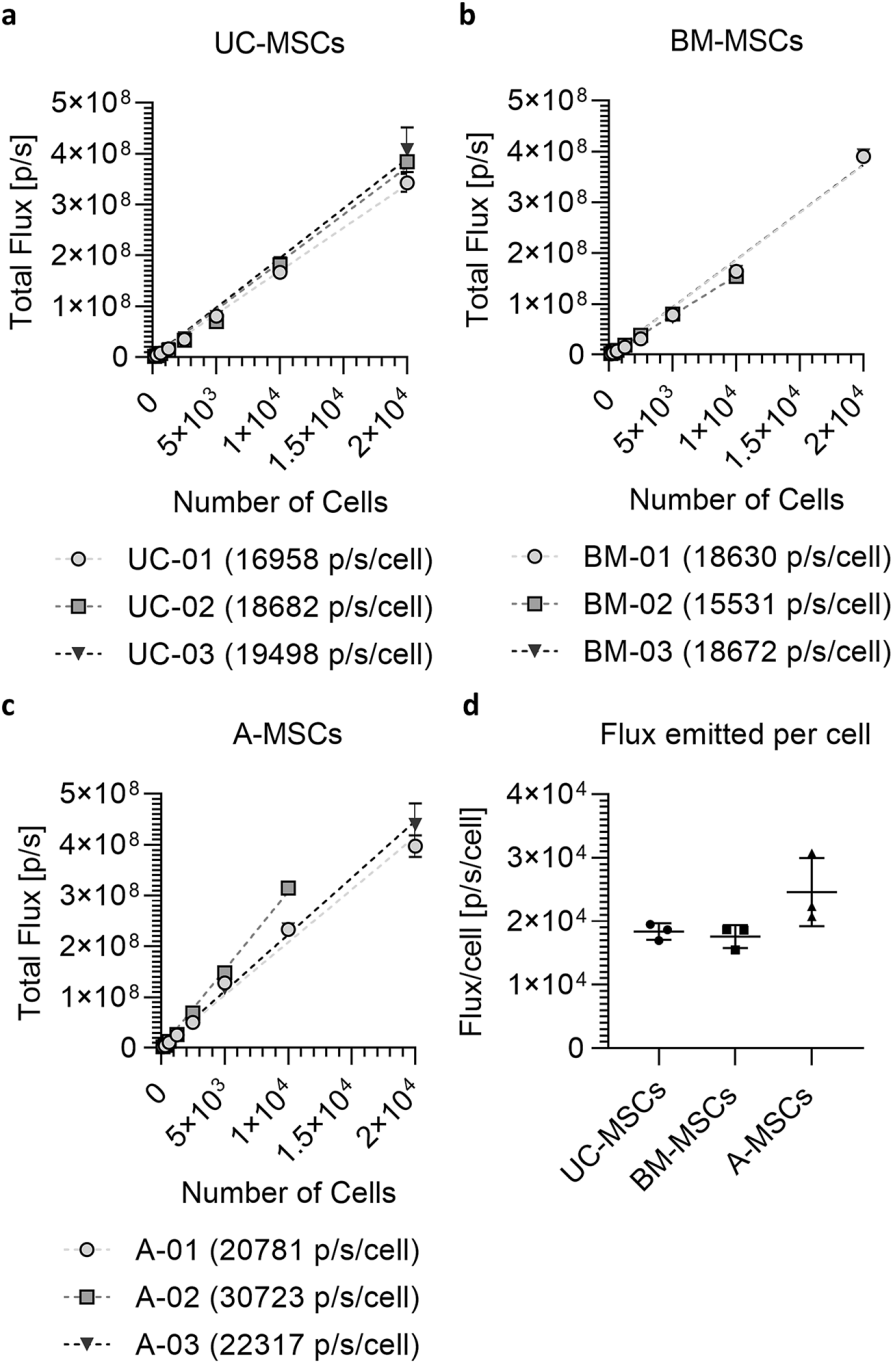
Light output of Luc2-ZsGreen^+^ MSCs as a function of cell density. Luc2-ZsGreen^+^ expressing MSCs were seeded at a density of 156 to 2 × 10^4^ cells/well and treated with saturating concentration of D-Luciferin (5.12 mM D-Luciferin. **a–c** Light output (flux) as a function of cell concentration, with linear regression curves of UC (**a**), BM (**b**) and A (**c**) MSCs for each individual donor sample. The slope of each curve represents the flux/cell and is shown in the legend of the respective graph. Data are displayed as mean ± SD from *n* = 3 replicates. Missing values are due to saturation of the signal (A-02) or unexpected dropping with 2 × 10^4^ cells (BM-02). **d** Average of the total amount of photon/second emitted by each cell type following transduction. Data obtained by averaging the results from individual donors. Data are displayed as mean ± SD from *n* = 3. One-way ANOVA with Tukey’s multiple comparison post hoc test

To measure the total flux emitted by each donor and confirm that the luciferase gene is also functional, cells were plated at various densities from 156 to 2 × 10^4^ cells/well and subsequently imaged in the presence of the D-Luciferin substrate, at a final concentration of 5.12 mM [5]. A linear regression of the signal measured revealed that the output from the three UC-MSC donor samples was similar (16,958 p/s/cell, 18,682 p/s/cell and 19,498 p/s/cell for UC-01, UC-02 and UC-03, respectively, Fig. 4a) and comparable to the BM-MSCs (18,630 p/s/cell, 15,531 p/s/cell and 18,672 p/s/cell for BM-01, BM-02 and BM-03, respectively, Fig. 4b). The output from the A-MSCs revealed one sample displaying higher signal when compared to the other two (20,781 p/s/cell, 30,723 p/s/cell and 22,317 p/s/cell for A-01, A-02 and A-03, respectively, Fig. 4c). On average, UC and BM-MSCs displayed very similar signal outputs (18,379 ± 1297 p/s/c and 17,611 ± 1,801 p/s/c, respectively), whilst A-MSCs resulted in a stronger signal (24,607 ± 5325 p/s/c), although no statistically significant difference was observed between MSCs from different tissue sources (Fig. 4d).

## Discussion

The purpose of this study was to identify a lentiviral transduction protocol suitable to infect human umbilical cord, bone marrow and adipose MSCs and generate stable cell populations without affecting their proliferation and properties.

Lentiviral transduction can be used to generate stable cell lines expressing a specific gene of interest [19]. However, the transduction with the LV alone is inefficient as shown both in the literature [12, 20] and in this study. There are reports showing that several polycations, like Pb [10, 11], Ps [15, 20] and DEAE-dextran [9], and the combination of these with a spinoculation step [16–18], can increase the transduction efficiency of different cells. Here we aimed at identifying which of these adjuvants is most effective to transduce MSCs from umbilical cord, bone marrow and adipose tissue, and whether spinoculation does indeed have a positive effect on the transduction of these cells.

For that, we tested the impact of different polycations (Pb, Ps and DEAE-dextran) on transduction efficiency and on cell proliferation, when applied alone or in combination with a centrifugation step. The transduction efficiency in the presence of Pb (8 µg/mL) was high for all the MSCs, with more than 90% of UC and BM cells transduced in both static and centrifuged conditions and with around 50% of adipose cells transduced in static condition. However, proliferation of the cells incubated with Pb in static condition was significantly reduced in comparison to controls. We observed this effect not only on the cells incubated with the lentiviral particles (32%, 72% and 74% for UC, BM and A-MSCs respectively), but also on the cells incubated just with the Pb overnight (56%, 69% and 78% for UC, BM and A-MSCs respectively). These results are in line with previous studies [12–14, 20] that reported an association between the presence of polybrene and a reduction in the proliferation of different cell types. Griukova et al.[14] has previously shown that Pb induces a p38 mitogen-activated protein kinase (MAPK) dependent premature senescence in human endometrial MSCs [14]. They reported enhanced β-galactosidase activity, a marker extensively used for the detection of senescence, and reduction in the expression of CD146, a novel marker for detecting senescence in UC-MSCs [14, 21]. Furthermore, Lin et al.[13] reported that even when using a potent mitogen, such as fibroblast growth factor 2, it is not possible to overcome the negative effects of Pb [13]. Lin et al. also reported a significant reduction in proliferation of human bone marrow MSCs with a polybrene concentration of 4 µg/mL, which is half of what was used here [13].

The p38 MAPK-dependent senescence might explain the reduced proliferation observed here when applying the Pb static condition, but we note that it was possible to reduce the negative effect of Pb on BM and A-MSCs with the use of the centrifugation protocol, which reduced the exposure to the lentiviral particles and polycation to only 2 h, although the impact on UC-MSCs was still substantial. Furthermore, the number of A-MSCs transduced with Pb following the introduction of the centrifugation step dropped from 48.6% to only 19.2%. For these reasons, we did not further investigate the use of Pb, as we wanted to identify a method suitable for the transduction of all the three MSC types. It is important to underline that the use of Pb together with a centrifugation step could be a viable option to transduce BM-MSCs, as it is associated with a very high transduction efficiency (> 94%) without impacting cell viability (94% of control).

As possible alternative protocols we identified: (a) the incubation with Ps coupled with centrifugation, as it was associated with a good transduction efficiency for UC and BM cells (70.7% and 75.8%, respectively) with a reduced impact on the proliferation of the cells (74% for UC and 119% for BM) or (b) the DEAE-dextran overnight incubation in static condition, as it was associated with a good transduction efficiency for all the cell types tested (74.4% for UC, 80.2% for BM and 50.0% for A-MSCs) without overtly impacting the proliferation of the cells (77% for UC, 116% for BM and 107% for A-MSCs). Because we were interested in a protocol suitable to transduce all the three cell types tested, and because the presence of centrifugation affected the viability of UC-MSCs, we opted for the DEAE-dextran static method. The superiority of the DEAE-dextran to Pb has been previously shown by Denning et al. [9], but their work was focussed on the 293FT and HT-1080 cell lines rather than primary MSCs. It should be noted that some studies have seen further enhancements in transduction efficiency with Ps by increasing its concentration to up to 100 µg/mL [15], although it seems that this is not consistent throughout the literature, with some investigators identifying a plateau at 20 µg/mL [20], the concentration we used here.

A similar analysis where different transduction protocols were tested to optimise the transduction of natural killer (NK) cells was carried out by Malarkannan et al. [22]. They investigated the transduction efficiency and the impact on cell viability following the lentiviral transduction of primary human and murine NK cells in the presence of either Pb (8 µg/mL), Ps (8 µg/mL) or DEAE-dextran (8 µg/mL) [22]. In this study, they combined a 1 h centrifugation step at 1000 g and an overnight incubation at 37 °C. Interestingly they obtained a strong increase in the transduction efficiency by incubating the cells with DEAE-dextran and almost no transduction with the two other polycations [22].

The data from Denning et al. [9] and Malarkannan and co-workers [22] suggest that the type of cell and the lentiviral particle itself might influence the outcome of the transduction and one single protocol might not be suitable for all cell types and LV constructs. Because of this, we investigated the DEAE-dextran static protocol for its ability to transduce the MSCs with four different lentiviruses, characterised by differences in their backbone and in the size of the insert. Regardless of the lentiviral construct used, the DEAE-dextran protocol enabled an increase in the transduction efficiency when compared to the cells incubated with the LVs alone. However, we identified a difference in the transduction efficiency of the different cells that was directly related to the nature of the lentiviral vector. This is expected, as several factors can influence not only the transduction efficiency but also the transgene expression level of a specific lentiviral vector. For instance, it has been previously shown that efficiency of transduction decreases with an increase in the size of the construct [23, 24]. In particular, Canté-Barrett et al. [24] reported that the transduction efficiency of human hematopoietic stem cells decreases significantly when the size of the insert in the LV was close to 6 kb or larger [24]. The results obtained in the present study are in line with those findings, as the plasmid with the largest insert, the pCDH-EF1α-Luc2-P2A-tdTomato (7046 bp insert size) was associated with a much lower transduction efficiency than the 3 other plasmids tested, which had smaller inserts (5038 bp, 5032 bp and 3938 bp for the pHIV-eGFP, the pHIV-dTomato and the pLV-mCherry, respectively). Furthermore, it was also previously reported that the gene sequence in the insert can affect the lentiviral infection [24]. This can further explain not only what was observed when applying the DEAE-dextran protocol with 4 different LVs, but also the high transduction efficiency observed when infecting the cells with the pHIV-Luc2-ZsGreen LV particles, which has an insert of 6663 bp.

Furthermore, the percentage of transduced cells for each lentiviral particle was significantly correlated with the tissue origin of the MSCs, confirming that the type of cell also has a role in the outcome of the transduction. For example, the percentage of A-MSCs transduced with the pHIV-Luc2-ZsGreen vector was much lower than the percentage of transduced UC or BM-MSCs. Interestingly we saw little variation between donor samples from the same tissue source, indicating that this is not likely to be a major variable when transduction efficiency is considered.

Finally, a critical aspect of this study was the production of transduced MSCs that can be used for further applications, without modifying or impairing the properties of the cells. Transduced cells should be as similar as possible to the naïve, non-genetically modified MSCs in order to be representative. We investigated this by assessing their proliferation, expression of MSC markers and morphology. We transduced a total of 9 donor samples (3 per cell type) using the selected protocol (6 µg/mL DEAE-dextran) and the pHIV_Luc2_ZsGreen LV with an MOI of 5, and we sorted the cells following the transduction to obtain pure FLuc expressing populations. We showed that all the transduced cells display similar properties to their untransduced counterparts in terms of doubling time, proliferation and expression of common MSC markers. However, it is interesting to point out that one UC-MSC displayed an area larger than the respective control after transduction, and that one A-MSC was slightly less circular than the respective control. Additionally, one A-MSC has an increased expression of CD45 after transduction, indicating the need to properly characterise the effect of transgene expression on cell properties, as this is no doubt dependent on the transgene.

The 9 donor samples, when genetically modified, exhibited comparable emission of light in the presence of D-Luciferin, underlining the replicability of the methodology. The average flux emitted ranged from 15,531 p/s/cell for one of the bone marrow samples to 30,723 p/s/cell for one of the adipose samples. These values are much higher than those described in a previous study, which reported that transduction of a mouse derived MSC cell line with the pHIV-Luc2-ZsGreen plasmid in the presence of polybrene resulted in 1508 p/s/cell [25], which is around 10 times lower than the values obtained here. Although this lower flux was already sufficient for in vivo bioluminescence imaging [25], greater light emissions can yield better imaging sensitivity.

In summary, an overnight incubation with 6 µg/mL DEAE-dextran and a LV with an MOI of 5 can be used universally to enhance the transduction of umbilical cord, bone marrow and adipose-derived MSCs with several lentiviral particles. Characterisation of transduced cells expressing Luc2_ZsGreen revealed no major effects of the transduction on their properties. Still, as mentioned previously, the efficiency of transduction is strongly correlated with the LV and with the cell type used. Because of this, we suggest that several polycations and protocols should be tested when optimising a transduction protocol for a specific cell type.

## Supplementary Information

Below is the link to the electronic supplementary material.

Supplementary Figure 1 Lentiviral vector backbones. (a-b) Schematic representation of the lentiviral vectors used to generate Luc2-ZsGreen+ cells (a) and to perform the validation of the DEAE-Dextran protocol (b) (TIF 1375 kb)

Supplementary Figure 2 Effect of polycations and centrifugation on UC-MSC transduction. (a) Representative phase contrast images of untransduced and transduced UC-MSCs in static condition. The images of the transduced cells are coupled with the green fluorescence channel to show the expression of the ZsGreen protein. Scale bar 200 µm. (b) Representative phase contrast images of untransduced and transduced UC-MSCs following the application of the centrifugation protocol. The images of the transduced cells are coupled with the green fluorescence channel to show the expression of the ZsGreen protein. Scale bar 200 µm. All fluorescence images acquired under the same acquisition conditions (TIF 6917 kb)

Supplementary Figure 3 Effect of polycations and centrifugation on BM-MSCs transduction. (a) Representative phase contrast images of untransduced and transduced bone marrow MSCs in static condition. The images of the transduced cells are coupled with the green fluorescence channel to show the expression of the ZsGreen protein. Scale bar 200 µm. (b) Representative phase contrast images of untransduced and transduced bone marrow derived MSCs following the application of the centrifugation protocol. The images of the transduced cells are coupled with the green fluorescence channel to show the expression of the ZsGreen protein. Scale bar 200 µm (TIF 6662 kb)

Supplementary Figure 4 Effect of polycations and centrifugation on A-MSCs transduction. (a) Representative phase contrast images of untransduced and transduced adipose derived MSCs in static condition. The images of the transduced cells are coupled with the green fluorescence channel to show the expression of the ZsGreen protein. Scale bar 200 µm. (b) Representative phase contrast images of untransduced and transduced adipose derived MSCs following the application of the centrifugation protocol. The images of the transduced cells are coupled with the green fluorescence channel to show the expression of the ZsGreen protein. Scale bar 200 µm (TIF 5878 kb)

Supplementary Figure 5 ATP assay of transduced and untransduced MSCs. (a-c) Data from UC (a), BM (b) and A-MSCs (c) separated by static or centrifuged protocols. Data are displayed as mean ± SD from n = 3 donors for each tissue source. Each donor sample was measured in triplicate and averaged. Three-way ANOVA with Dunnett post-hoc comparison test against untransduced static control; * *p* < 0.05; ** *p* < 0.01; # *p* < 0.0005; $ *p* < 0.0001 (TIF 1139 kb)

Supplementary Figure 6 The application of the DEAE-dextran static protocol increases the transduction efficiency of all types of MSCs with all the lentiviral particles tested. (a-c) representative fluorescence images of UC- (a), BM- (b) and A- (c) MSCs transduced with four different lentiviral particles with and without the use of DEAE-dextran as a polycation. Scale bar 200 µm. Contrast in the images from eGFP cells was enhanced to facilitate the identification of transduced cells (TIF 5547 kb)

Supplementary Figure 7 Luc2-ZsGreen+ MSCs display similar proliferation to untransduced cells. (a) Cumulative doublings from p7 to p10 (UC-02 and UC-03) and from p7 to p11 (UC-01) for the 3 UC-MSC and respective controls. (b) Cumulative doubling from p5 to p7 (BM-01 and BM-03) and from p7 to p10 (BM-02) of the 3 BM MSC and respective controls. (c) Cumulative doubling from p7 to p9 (A-02) and p7 to p10 (A-01 and A-03) of the 3 A-MSC samples and respective controls. (d) Average doubling time of transduced and untransduced cells displayed by tissue of origin. FLuc = Luc2-ZsGreen+ cells. Data are displayed as mean ± SD, *n* ≥ 2 independent experiments. Two-way ANOVA analysis with Sidak’s multiple comparisons post-hoc test (TIF 1558 kb)

Supplementary Figure 8 Morphological characterisation of transduced MSCs. (a-b) Violin plot of the area (a) and the circularity (b) of the transduced and untransduced UC-MSC populations (at least 55 cells from each sample were analysed). (c-d) Violin plot of the area (c) and the circularity (d) of the transduced and untransduced BM-MSC populations (at least 60 cells for each sample were analysed). (e-f) Violin plot of the area (e) and the circularity (f) of the transduced and untransduced A-MSC populations (at least 64 cells for each sample were analysed). For all the graphs, the data were plotted into a grouped graph and cleaned from the outliers using the automated GraphPad tool “remove outliers” using the ROUT method with a Q = 1%. A two-way ANOVA was performed on the cleaned data with a Dunn’s multiple comparison post-hoc test; * *p* < 0.05, **** *p* < 0.0001 (TIF 1548 kb)

## Data Availability

All datasets from this study are publicly available on Zenodo, https://doi.org/10.5281/zenodo.6594236
