# Supplementary figures and images for: DEAE-Dextran Enhances the Lentiviral Transduction of Primary Human Mesenchymal Stromal Cells from All Major Tissue Sources Without Affecting Their Proliferation and Phenotype
Source: Mol Biotechnol. 2022 Aug 23;65(4):544–55. doi: 10.1007/s12033-022-00549-2 (PMC9974715; doi:10.1007/s12033-022-00549-2)

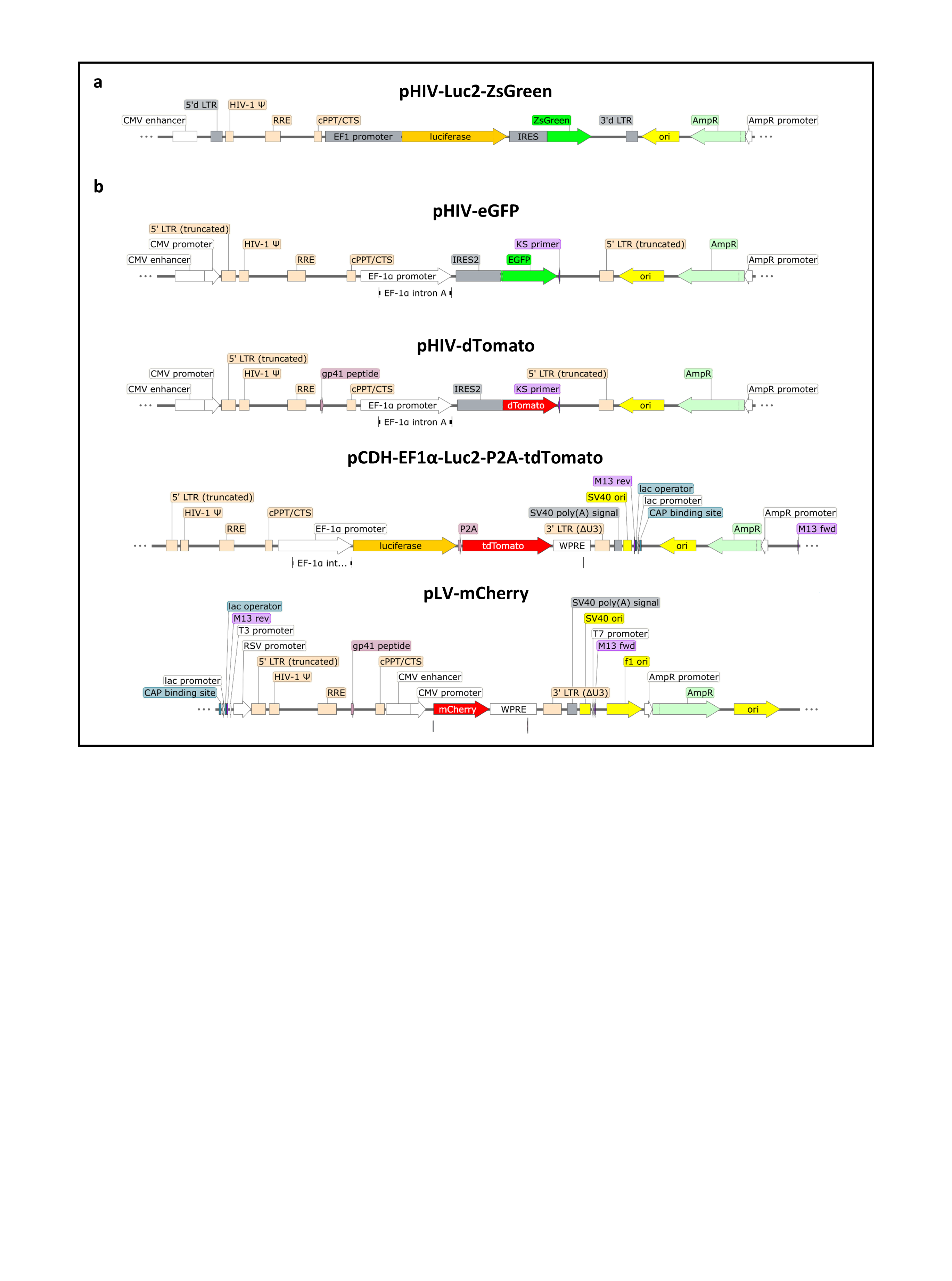

Supplement: Supplementary file 1 — Supplementary Figure 1 Lentiviral vector backbones. (a-b) Schematic representation of the lentiviral vectors used to generate Luc2-ZsGreen+ cells (a) and to perform the validation of the DEAE-Dextran protocol (b) (TIF 1375 kb) [file 12033_2022_549_MOESM1_ESM.tif]

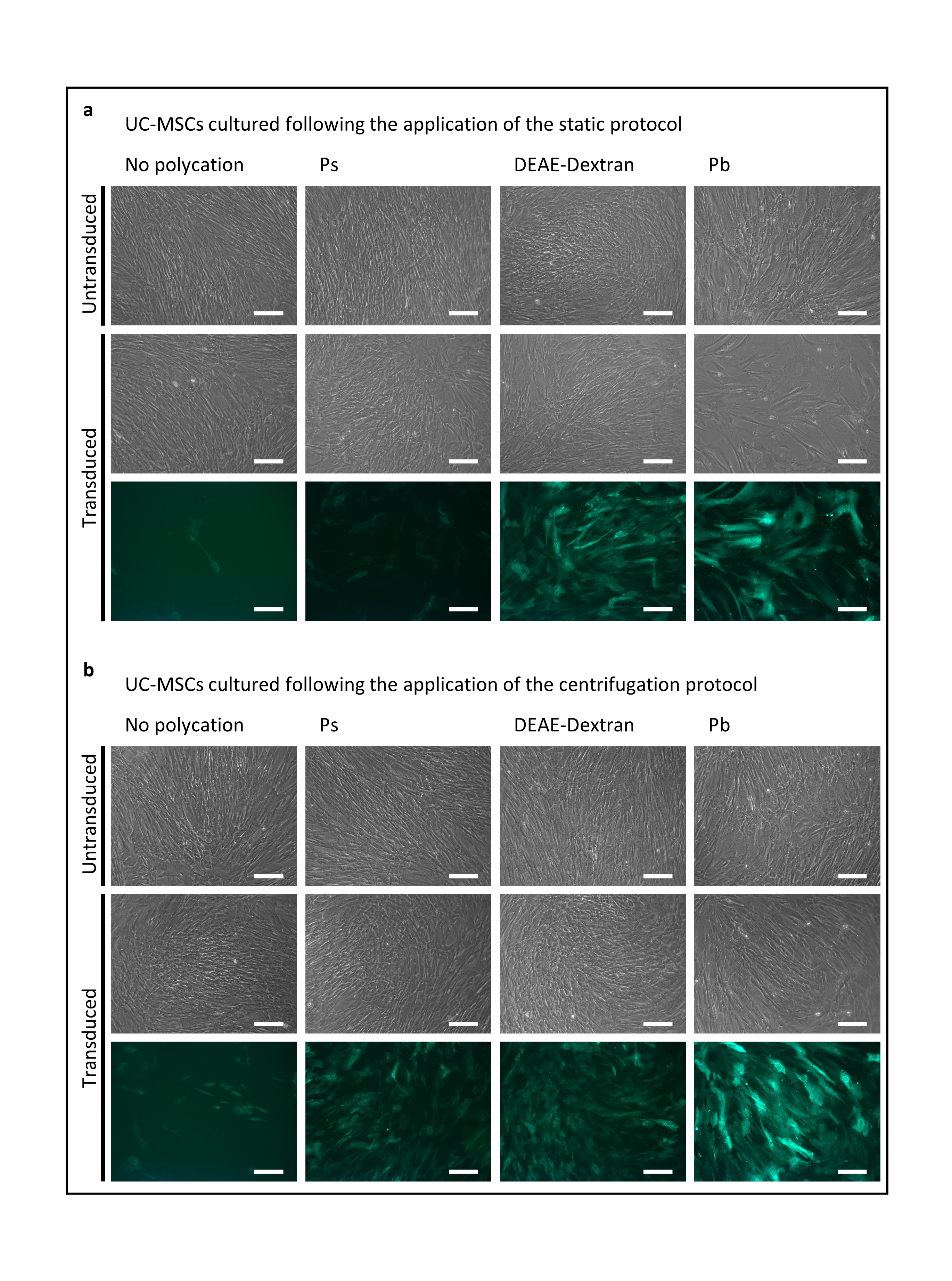

Supplement: Supplementary file 2 — Supplementary Figure 2 Effect of polycations and centrifugation on UC-MSC transduction. (a) Representative phase contrast images of untransduced and transduced UC-MSCs in static condition. The images of the transduced cells are coupled with the green fluorescence channel to show the expression of the ZsGreen protein. Scale bar 200 µm. (b) Representative phase contrast images of untransduced and transduced UC-MSCs following the application of the centrifugation protocol. The images of the transduced cells are coupled with the green fluorescence channel to show the expression of the ZsGreen protein. Scale bar 200 µm. All fluorescence images acquired under the same acquisition conditions (TIF 6917 kb) [file 12033_2022_549_MOESM2_ESM.tif]

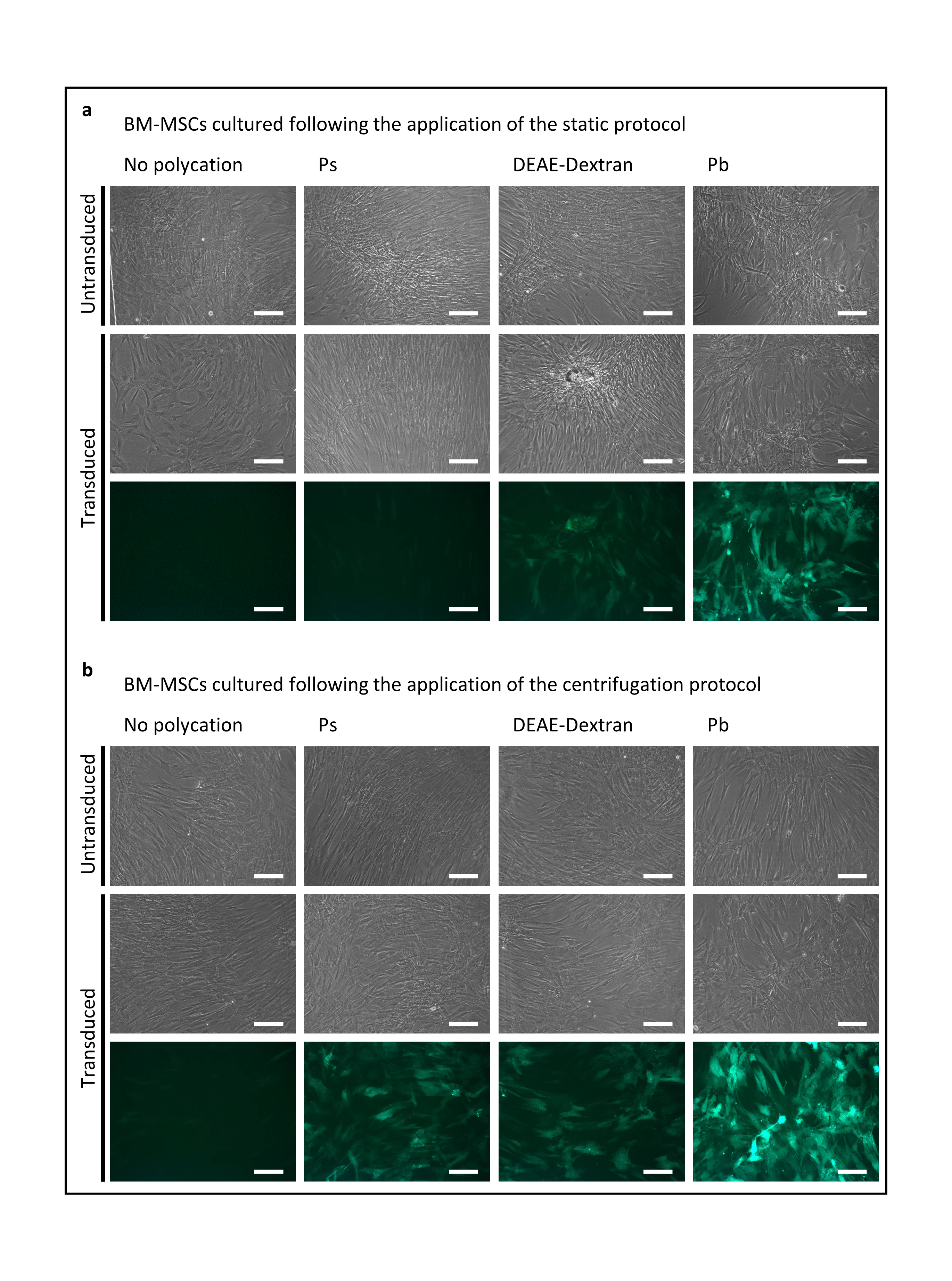

Supplement: Supplementary file 3 — Supplementary Figure 3 Effect of polycations and centrifugation on BM-MSCs transduction. (a) Representative phase contrast images of untransduced and transduced bone marrow MSCs in static condition. The images of the transduced cells are coupled with the green fluorescence channel to show the expression of the ZsGreen protein. Scale bar 200 µm. (b) Representative phase contrast images of untransduced and transduced bone marrow derived MSCs following the application of the centrifugation protocol. The images of the transduced cells are coupled with the green fluorescence channel to show the expression of the ZsGreen protein. Scale bar 200 µm (TIF 6662 kb) [file 12033_2022_549_MOESM3_ESM.tif]

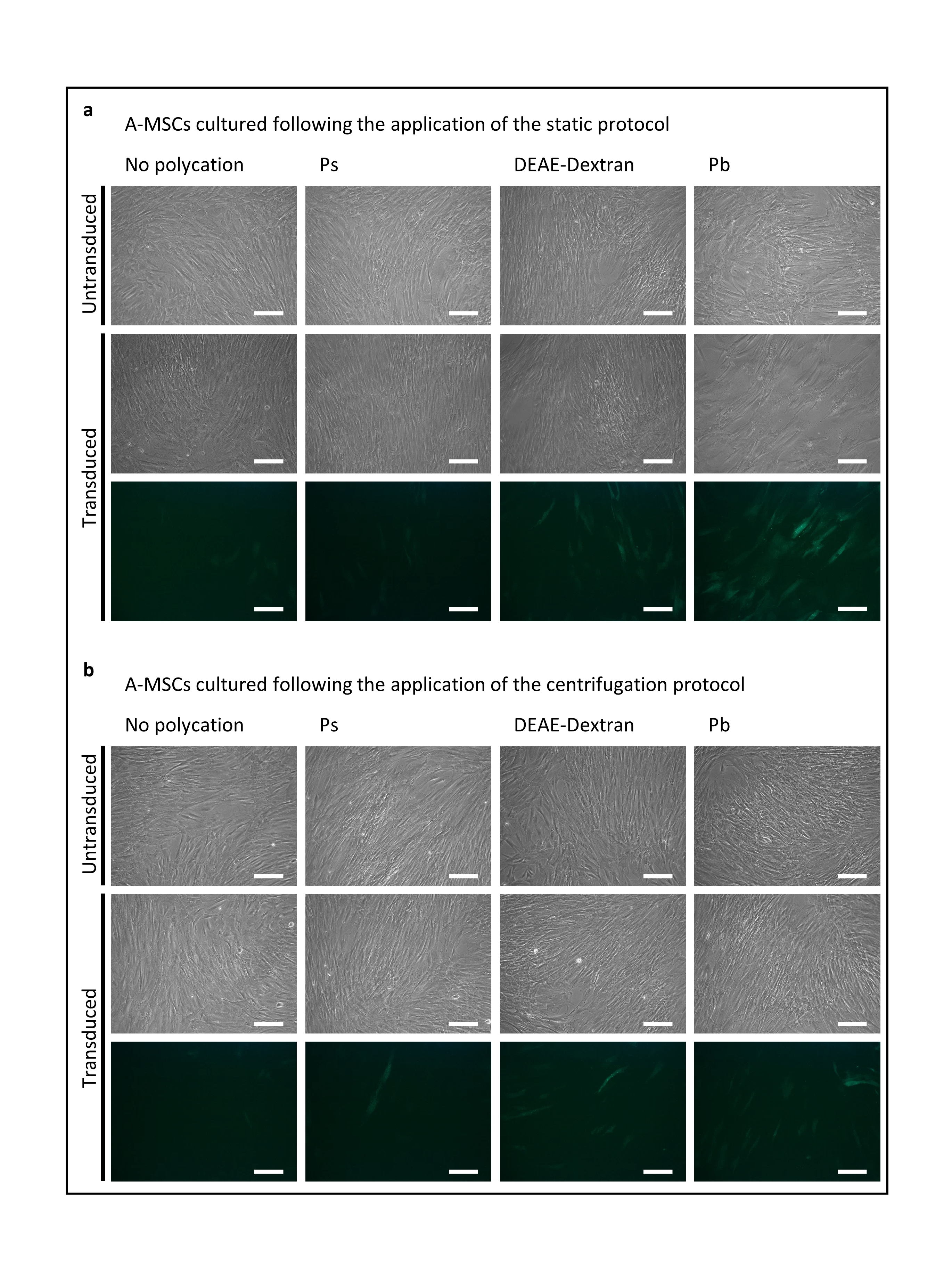

Supplement: Supplementary file 4 — Supplementary Figure 4 Effect of polycations and centrifugation on A-MSCs transduction. (a) Representative phase contrast images of untransduced and transduced adipose derived MSCs in static condition. The images of the transduced cells are coupled with the green fluorescence channel to show the expression of the ZsGreen protein. Scale bar 200 µm. (b) Representative phase contrast images of untransduced and transduced adipose derived MSCs following the application of the centrifugation protocol. The images of the transduced cells are coupled with the green fluorescence channel to show the expression of the ZsGreen protein. Scale bar 200 µm (TIF 5878 kb) [file 12033_2022_549_MOESM4_ESM.tif]

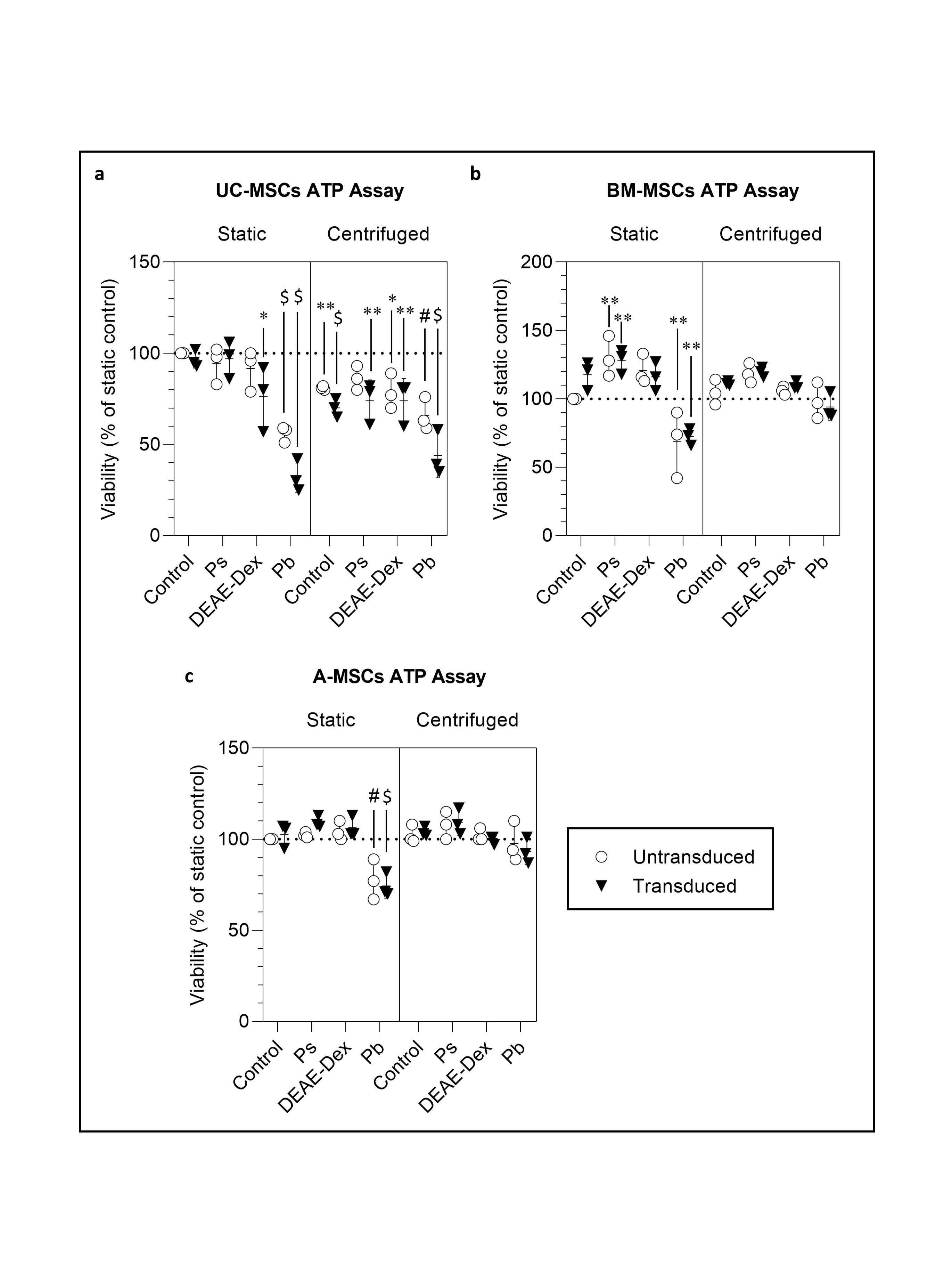

Supplement: Supplementary file 5 — Supplementary Figure 5 ATP assay of transduced and untransduced MSCs. (a-c) Data from UC (a), BM (b) and A-MSCs (c) separated by static or centrifuged protocols. Data are displayed as mean ± SD from n = 3 donors for each tissue source. Each donor sample was measured in triplicate and averaged. Three-way ANOVA with Dunnett post-hoc comparison test against untransduced static control; * p < 0.05; ** p < 0.01; # p < 0.0005; $ p < 0.0001 (TIF 1139 kb) [file 12033_2022_549_MOESM5_ESM.tif]

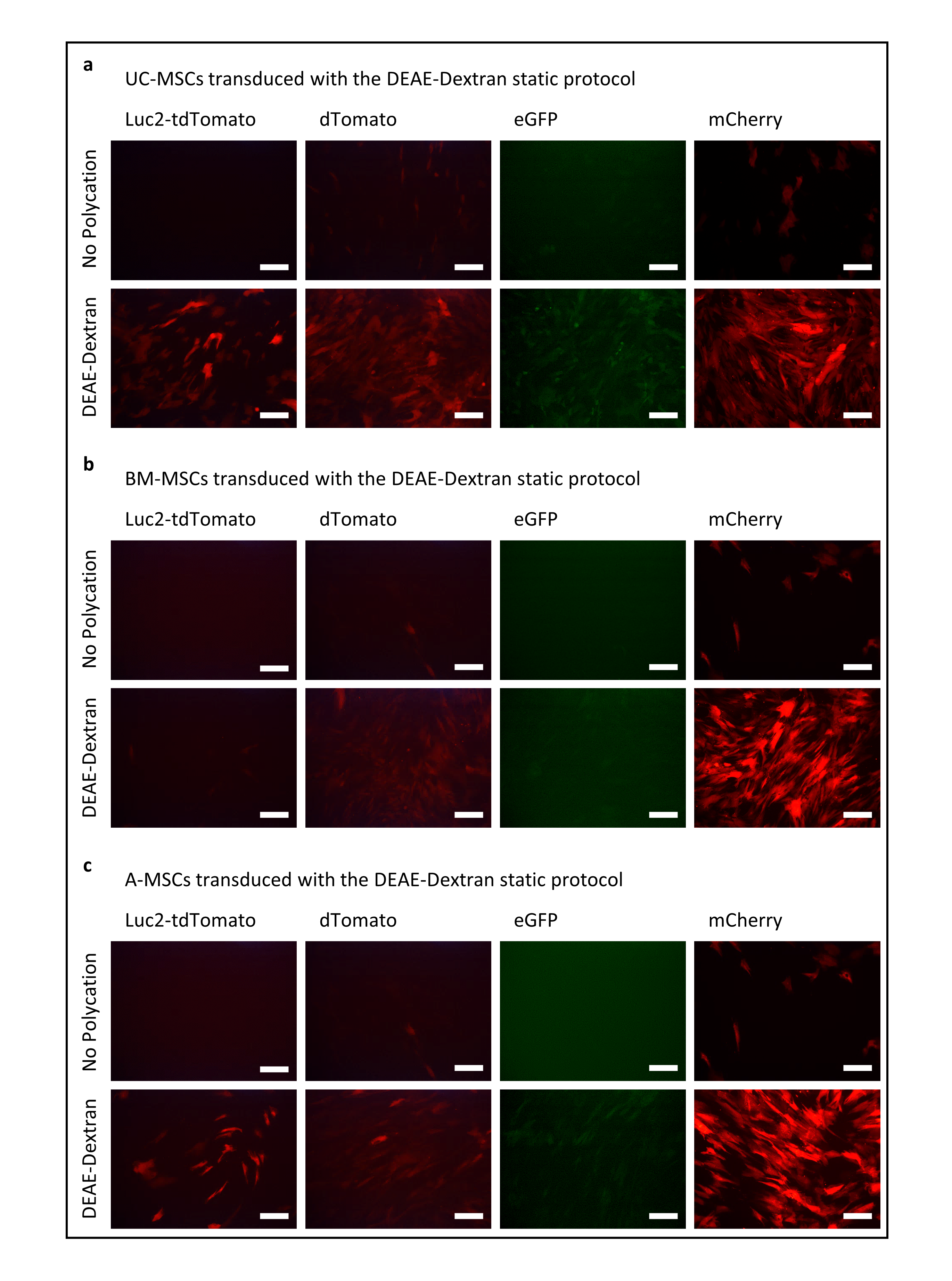

Supplement: Supplementary file 6 — Supplementary Figure 6 The application of the DEAE-dextran static protocol increases the transduction efficiency of all types of MSCs with all the lentiviral particles tested. (a-c) representative fluorescence images of UC- (a), BM- (b) and A- (c) MSCs transduced with four different lentiviral particles with and without the use of DEAE-dextran as a polycation. Scale bar 200 µm. Contrast in the images from eGFP cells was enhanced to facilitate the identification of transduced cells (TIF 5547 kb) [file 12033_2022_549_MOESM6_ESM.tif]

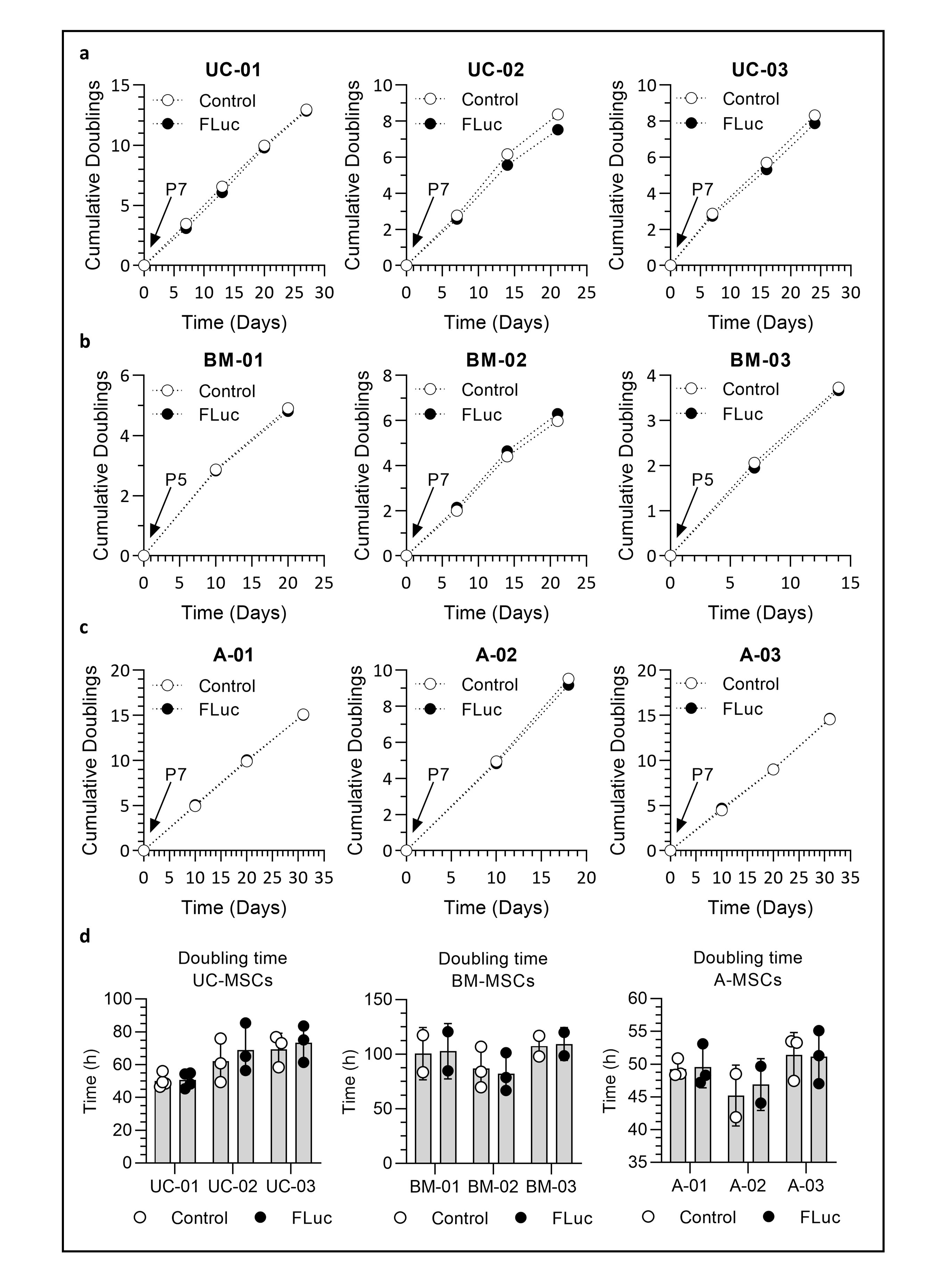

Supplement: Supplementary file 7 — Supplementary Figure 7 Luc2-ZsGreen+ MSCs display similar proliferation to untransduced cells. (a) Cumulative doublings from p7 to p10 (UC-02 and UC-03) and from p7 to p11 (UC-01) for the 3 UC-MSC and respective controls. (b) Cumulative doubling from p5 to p7 (BM-01 and BM-03) and from p7 to p10 (BM-02) of the 3 BM MSC and respective controls. (c) Cumulative doubling from p7 to p9 (A-02) and p7 to p10 (A-01 and A-03) of the 3 A-MSC samples and respective controls. (d) Average doubling time of transduced and untransduced cells displayed by tissue of origin. FLuc = Luc2-ZsGreen+ cells. Data are displayed as mean ± SD, n ≥ 2 independent experiments. Two-way ANOVA analysis with Sidak’s multiple comparisons post-hoc test (TIF 1558 kb) [file 12033_2022_549_MOESM7_ESM.tif]

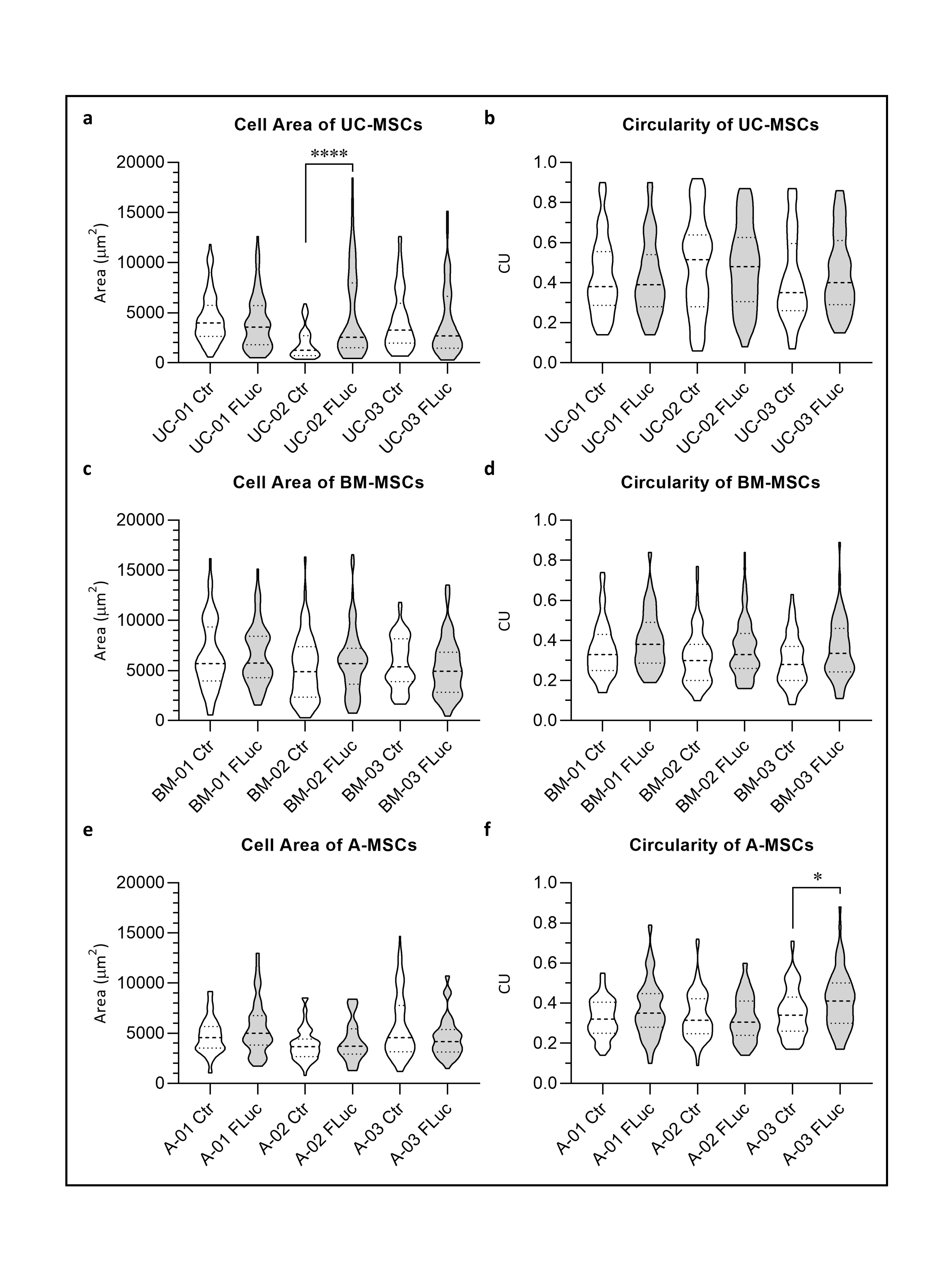

Supplement: Supplementary file 8 — Supplementary Figure 8 Morphological characterisation of transduced MSCs. (a-b) Violin plot of the area (a) and the circularity (b) of the transduced and untransduced UC-MSC populations (at least 55 cells from each sample were analysed). (c-d) Violin plot of the area (c) and the circularity (d) of the transduced and untransduced BM-MSC populations (at least 60 cells for each sample were analysed). (e-f) Violin plot of the area (e) and the circularity (f) of the transduced and untransduced A-MSC populations (at least 64 cells for each sample were analysed). For all the graphs, the data were plotted into a grouped graph and cleaned from the outliers using the automated GraphPad tool “remove outliers” using the ROUT method with a Q = 1%. A two-way ANOVA was performed on the cleaned data with a Dunn’s multiple comparison post-hoc test; * p < 0.05, **** p < 0.0001 (TIF 1548 kb) [file 12033_2022_549_MOESM8_ESM.tif]
